# Does a Restrictive Diagnostic Work‐up for Thyroid Nodules Lead to a Different Papillary Thyroid Cancer Patient Population? A Comparison Between Dutch and U.S. T1‐T3 Patient Population

**DOI:** 10.1002/wjs.12457

**Published:** 2024-12-25

**Authors:** Maaike B. C. ten Hoor, Jia F. Lin, Madelon J. H. Metman, Pedro M. Rodriguez Schaap, Thera P. Links, Renske Altena, Tessa M. van Ginhoven, Wouter T. Zandee, Anton F. Engelsman, Schelto Kruijff

**Affiliations:** ^1^ Department of Surgical Oncology University Medical Center Groningen University of Groningen Groningen the Netherlands; ^2^ Department of Surgery Amsterdam University Medical Centre Location VUmc Cancer Centre Amsterdam Amsterdam the Netherlands; ^3^ Department of Internal Medicine Division of Endocrinology University Medical Center Groningen University of Groningen Groningen the Netherlands; ^4^ Karolinska Comprehensive Cancer Center Karolinska University Hospital Stockholm Sweden; ^5^ Institution for Oncology‐Pathology Karolinska Institutet Stockholm Sweden; ^6^ Department of Nuclear Medicine Function Medical Diagnostics Karolinska University Hospital Stockholm Sweden; ^7^ Department of Surgery Erasmus Medical Center Erasmus University Rotterdam The Netherlands; ^8^ Department of Nuclear Medicine and Molecular Imaging University Medical Center Groningen University of Groningen Groningen the Netherlands; ^9^ Department of Molecular Medicine and Surgery Karolinska Institutet Stockholm Sweden

**Keywords:** 2015 ATA guidelines, de‐escalation, papillary thyroid carcinoma, thyroid cancer, treatment

## Abstract

**Introduction:**

The 2015 American Thyroid Association guidelines recommend de‐escalating surgical treatment for papillary thyroid cancer (PTC). We hypothesize that the Dutch PTC population might differ due to a restrictive diagnostic policy that mainly selects symptomatic and palpable thyroid nodules for further diagnostics, potentially selecting relatively more aggressive tumors. We aimed to describe the Dutch PTC population because differences in populations can have consequences for the adoption of foreign guidelines.

**Methods:**

From the Dutch national cancer registry, patients diagnosed with pT1–T3 PTC between 2005 and 2015 were included. Baseline characteristics, disease‐free interval, and overall survival were compared between low‐risk and non‐low risk PTC. Furthermore, the TNM stage of the Dutch and U.S. cohorts were compared via literature search.

**Results:**

Of the 3368 pT1‐T3 patients included, 1813 (53.8%) had a low‐risk PTC, and 1555 (46.2%) had a non‐low‐risk PTC. In the Dutch PTC population, pT1 tumors occurred in 45.8%, pT2 and pT3 tumors occurred in 34.9% and 19.3% of the patients, respectively. Of all patients, 10.2% had central lymph node metastases and 16.6% had lateral lymph node metastasis. Distant metastasis only occurred in 18 (0.5%) of the patients. The 10‐year overall survival was 89.6%, with rates of 91.6% for low‐risk and 87.3% for non‐low‐risk patients (*p* = < 0.001). During the follow‐up period, 257 patients (7.6%) had a recurrence.

**Discussion:**

The higher frequency of advanced tumors among the Dutch PTC population in contrast to the U.S. emphasizes the need for careful national data analyses before the adoption of surgical intervention de‐escalation protocols from other countries.

## Graphical abstract

Does a restrictive diagnostic work‐up for thyroid nodules lead to a different papillary thyroid cancer patient population? A comparison between Dutch and U.S. PT1–T3 patient population.

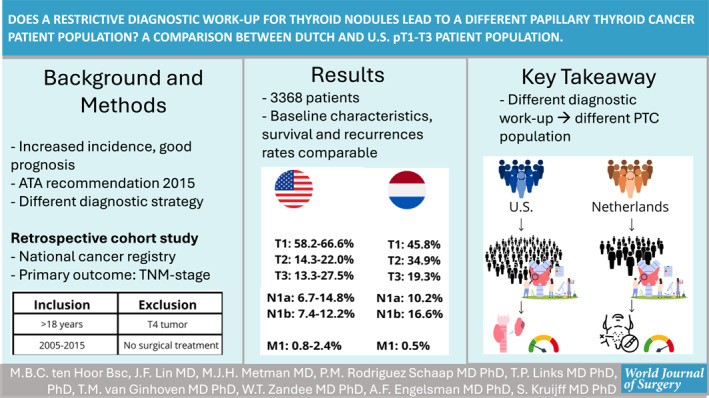

## 1 INTRODUCTION

Papillary thyroid cancer (PTC) is the most common thyroid malignancy, representing over 85% of thyroid cancer cases.^1^ The rising incidence of PTC is largely attributed to the increased utilization of imaging modalities.^2^ The 10‐year overall survival rate of 97% of PTC suggests that the surge in incidence primarily stems from overdiagnosis.^3^, ^4^ To prevent overtreatment, there has been a shift in the 2015 American Thyroid Association (ATA) guidelines toward a less aggressive surgical treatment for low‐risk PTC patients. This de‐escalation approach entails the recommendation to perform a hemithyroidectomy for 1–4 cm low‐risk PTCs instead of a total thyroidectomy followed by radioactive iodine (RAI).^5^

Since 2007, ultrasound‐guided fine needle aspiration (FNA) is only recommended for patients with a clinically palpable thyroid nodules in the Netherlands.^6^, ^7^ Consequently, incidental thyroid nodules on CT or MRI are rarely evaluated.^7^ Therefore, the Dutch guidelines only recommended a hemithyroidectomy for unifocal PTC smaller than 1 cm without indications of lymph node metastases, extrathyroidal extension, or an aggressive variant.^6^ In all other cases, a total thyroidectomy is performed, usually followed by RAI therapy.^6^ Selective neck dissections were only performed in the case of clinical manifest lymph node metastases proven with FNA and was not done routinely.^6^

There is limited documentation regarding the influence of a more restrictive diagnostic work‐up on a national PTC population. The characteristics of patients that are eventually diagnosed with thyroid cancer and are offered treatment may differ among countries.^7^, ^8^ For example, in Belgium, regional differences in diagnostic practices were inversely related to thyroid cancer incidence and surgery rates.^9^ We hypothesize that the Dutch PTC population differs with the U.S. population due to a restrictive work‐up policy that only selects palpable thyroid nodules for further diagnostics, potentially leading to the selection of relatively more advanced tumors.^7^

In this study, we aim to outline the characteristics of the Dutch pT1–T3 PTC population. Moreover, we compare our population to the U.S. PTC cohorts, investigating if it is justified to copy guidelines from other countries, especially when these countries have a different diagnostic approach, which is the case with the 2015 ATA recommendation to de‐escalate surgical treatment.

## 2 PATIENTS AND METHODS

### 2.1 Study design and participants

This retrospective cohort study was performed at the Surgical Oncology department of the University Medical Center Groningen. Data from 2005 until 2015 was collected and obtained from the database of the Netherlands Comprehensive Cancer Organization (IKNL), the national cancer registry. This was linked to data from the National Network and Registry of Histo‐ and Cytopathology in the Netherlands (PALGA) via de‐identified patient numbers. The IKNL database provided clinical variables such as sex, age at diagnosis, vital status, follow‐up duration, and RAI therapy (RAI). Pathology reports from the PALGA database were reviewed, and patients were reclassified according to the eighth edition of the American Joint Committee on Cancer ( of the American Joint Committee on Cancer) TNM staging criteria.^10^ We included patients aged 18 years or older at the time of PTC diagnosis, excluding those with pT4 tumors.^5^ Subsequently, patients were categorized into two groups: low‐risk and non‐low‐risk PTC.^5^ Low‐risk tumors were characterized according to the 2015 ATA risk stratification: <5 positive lymph nodes in the central compartment, absence of distant metastasis, lack of aggressive histology, and no vascular invasion.^5^ Aggressive histology was defined as tall cell, diffuse sclerosing, columnar cell, or Hürthle cell PTC.^5^ All other patients were classified as non‐low‐risk when they exhibited one or more of the following: gross extrathyroidal extension, pT3 tumor, distant metastasis, incomplete tumor resection, lateral lymph node metastasis, and vascular invasion. A total thyroidectomy is defined as the total thyroidectomies or hemithyroidectomy followed by completion hemithyroidectomy within 12 months.

### 2.2 Follow‐up, recurrence, and overall survival

Vital follow‐up continued until December 2019, with patient status verified through the IKNL database and personal records database (BRP). Overall survival was defined as the time between PTC diagnosis and death from any cause. Recurrence was determined using PALGA data and defined as a histologically or cytologically proven locoregional recurrence or distant metastasis occurring at least 12 disease‐free months post‐surgery. The disease‐free interval (DFI) was the period from primary surgery until recurrence.

### 2.3 Comparison data

A PubMed literature search on PTC population cohorts from the U.S. was performed from inception to May 16, 2024. Search terms included controlled terms and free text terms, focusing on studies based on the U.S. Surveillance Epidemiology and End Results (SEER) database. Initially, the search targeted the ratio between low‐, intermediate‐, and high‐risk PTC, but due to limited results (see Supplementary Table 1), it was refined to compare the TNM stage between PTC populations with the terms, SEER and PTC, yielding 237 results. Titles were screened first, followed by abstracts, and then full manuscripts (Supplementary Table 2).

### 2.4 Statistical analyses

Descriptive statistics were presented as means with standard deviation (SD) or medians with interquartile range (IQR) for non‐normally distributed data. The normal distribution of continuous variables was assessed using Q–Q plots. Normally distributed variables were compared using the *t*‐test and non‐normally distributed variables using the Mann–Whitney *U*‐test. Categorical variables were presented as percentages and compared using the Chi‐Square test. Overall survival and DFI were estimated using Kaplan–Meier curves, and differences between groups were assessed using the log‐rank test. The univariate analysis of factors associated with death from any cause and recurrence was performed using Cox regression analysis for the overall, low‐risk, and non‐low‐risk group. Significant factors were analyzed per group using multivariable Cox regression analysis.

## 3 RESULTS

### 3.1 Patient inclusion and study population characteristics

**FIGURE 1:**
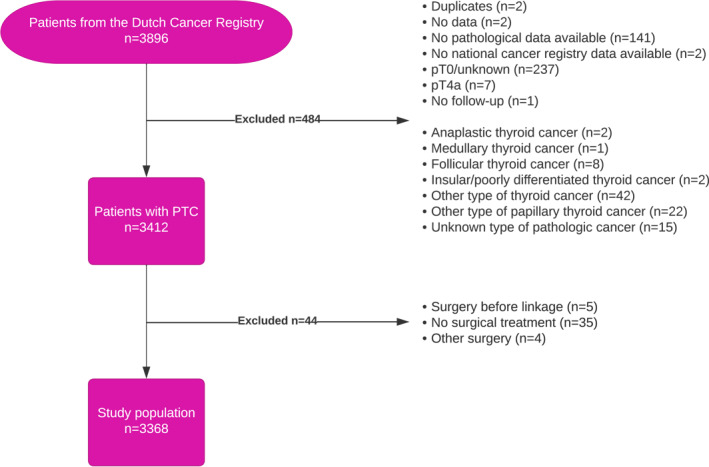
Flowchart of the inclusion and exclusion criteria resulting in the study population. Abbreviation: PTC, papillary thyroid cancer.

Between 2005 and 2015, 3896 patients with pT1‐T3 PTC underwent surgical treatment in the Netherlands. After excluding 528 patients who did not meet the inclusion criteria (see Figure 1), 3368 patients were included in the study (Table 1). The cohort consisted of 2501 females (74.3%) and 867 males (25.7%), with a mean age at diagnosis of 48.7 years (SD ± 15.1), and the median vital follow‐up was 5.8 years (IQR: 3.7–8.7). Of all patients, 1813 (53.8%) had a low‐risk tumor. Notably, a higher proportion of males (32.5%) had a non‐low‐risk PTC compared to females (19.9%) (*p* < 0.001). Postoperative RAI therapy was administered to 2514 (74.6%) patients, with a significant higher rate among those with non‐low‐risk PTC (low‐risk: 61.7% vs. non‐low‐risk: 89.7%) (*p* < 0.001).

**TABLE 1:** Dutch national registry population characteristics.

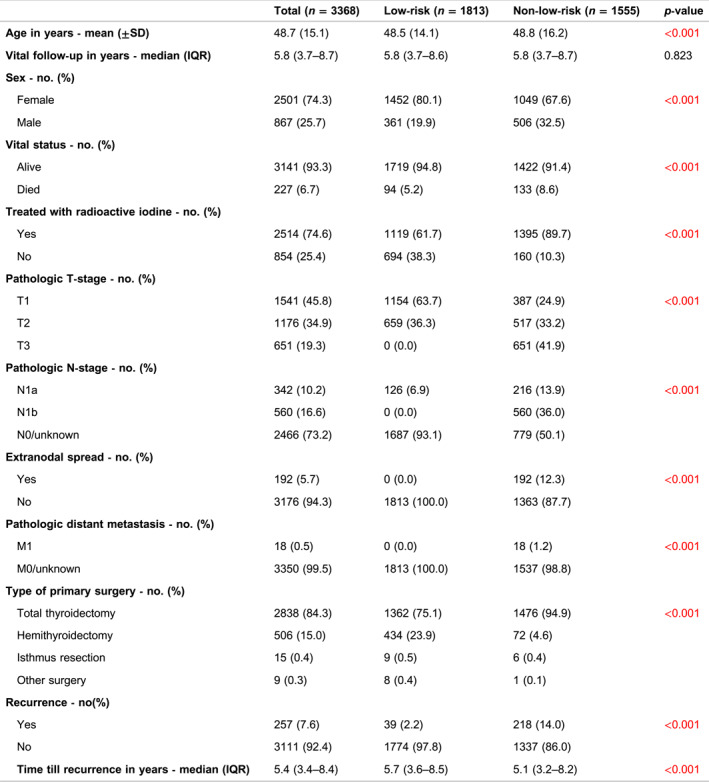

### 3.2 Surgery

2838 patients underwent a total thyroidectomy (84.3%). Of these, 1434 (42.6%) underwent total thyroidectomy initially, while 1404 patients (41.7%) underwent hemithyroidectomy followed by complementary surgery. Hemithyroidectomy alone was performed in 15.0% of the cases, and an isthmus resection was performed in 0.4% (*n* = 15). Other surgeries included median neck cyst removal (*n* = 6), thyroglossal duct cyst removal (*n* = 2), and pyramidal lobe removal (*n* = 2). As expected, total thyroidectomy was performed more in the non‐low‐risk group compared to the low‐risk group (94.4% vs. 75.1%). Hemithyroidectomy was performed in 23.9% of the low‐risk patients and 4.6% of the non‐low‐risk patients. Additionally, a lateral neck dissection during primary surgery was performed in 11.7% (9.7% unilateral, 2.0% bilateral) of the patients.

### 3.3 Histology

The classic type of PTC was most prevalent, accounting for 2367 (70.3%) of the patients, followed by the follicular variant (22.5%) and mixed papillary and follicular (5.8%). Less common were the more aggressive types of PTC, including tall cell (0.4%), diffuse sclerosing (0.3%), columnar cell (0.1%), and Hürthle cell (0.5%) (see Table 2). Vascular invasion was present in 11.3% of the patients, and the prevalences of R0, R1, and R2 in the total group were 82.5%, 9.0%, and 8.6%, respectively. Multifocal disease was seen in 37.5% of patients, occurring more frequently in the non‐low‐risk group (44.1% vs. 31.9%, *p* < 0.001). The median tumor size in the low‐risk group was 13.0 mm (IQR: 6.0–22.0), significantly smaller when compared to the non‐low‐risk group (25.0 mm, IQR: 15.0–45.0) (*p* < 0.001). Furthermore, tumors were bilaterally located in 22.4% of all patients, with a higher frequency observed in the non‐low‐risk group (27.7 vs. 17.8%, *p* < 0.001).

**TABLE 2:** Pathology characteristics of Dutch papillary thyroid cancer patients.

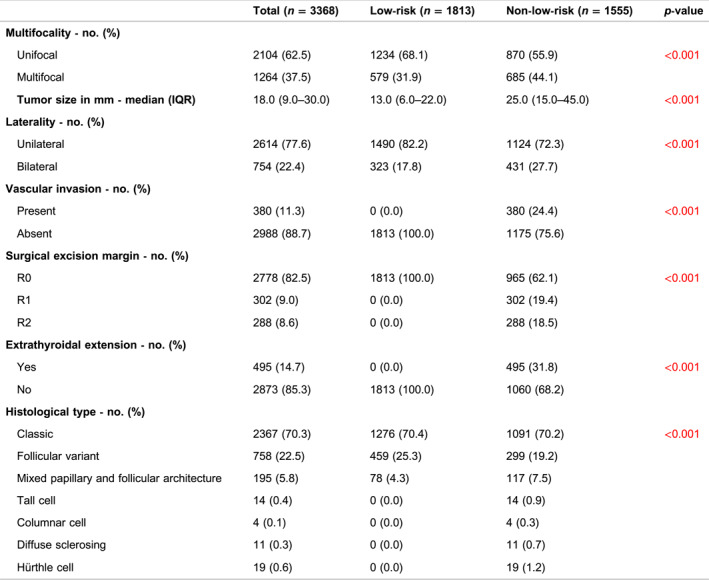

### 3.4 TNM stage

Pathological TNM staging data is provided in Table 1. The prevalences of pT1, pT2, and pT3 tumors were 45.8%, 34.9%, and 19.3%, respectively. Nodal metastases were present in 902 patients (26.8%), with 126 cases (6.9%) of central nodal metastases observed in the low‐risk group. Distant metastases at diagnosis were detected in 18 patients (0.5%), primarily affecting the lungs (*n* = 9), bones (*n* = 6), and isolated cases in the brain, thoracic wall, and thymus. pT1 tumors were most common (63.7%) in patients with low‐risk PTC, whereas pT3 tumors were predominant (41.9%) in the non‐low‐risk group (*p* < 0.001).

### 3.5 Overall survival

**FIGURE 2:**
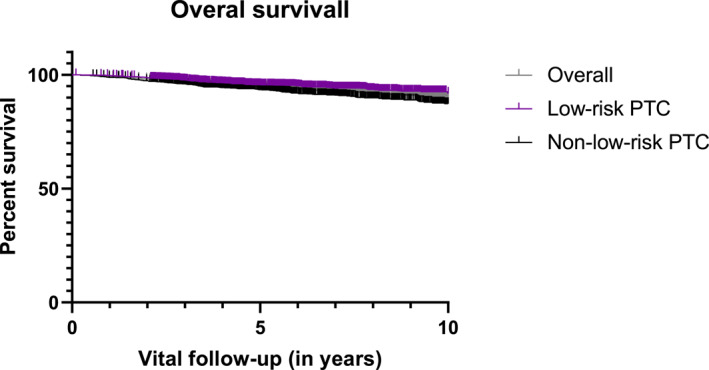
The 10‐year overall survival in the low‐risk and non‐low‐risk Dutch papillary thyroid cancer patients.

The median vital follow‐up was 5.8 years during which 3141 (93.3%) patients remained alive (Table 1). Mortality from any cause was more frequent in the non‐low‐risk group compared to the low‐risk group (5.2% vs. 8.6%, *p* < 0.001). The 10‐year overall survival for all patients was 89.6%, with rates of 91.6% for low‐risk patients and 87.3% for non‐low‐risk patients (*p* < 0.001) (Figure 2). Multivariable Cox regression analysis revealed that only male sex (hazard ratio: 1.6; 95% confidence interval: 1.2–2.1; *p* < 0.001), age >55 years (HR: 9.9; 95% CI: 7.0–13.8; *p* < 0.001), total thyroidectomy (HR: 1.7; 95% CI: 1.2–2.3; *p* = 0.002), RAI therapy (HR: 0.5; 95% CI: 0.4–0.7; *p* < 0.001), pT3 stage (HR: 1.5; 95% CI: 1.1–2.1; *p* = 0.20), lateral lymph node metastasis (HR: 2.0; 95% CI: 1.3–2.9; *p* < 0.001), and distant metastasis at diagnosis (HR: 3.6; 95% CI: 2.0–6.6; *p* < 0.001) remained independently associated with death from any cause (Table 3). In the low‐risk group, male sex (HR: 2.0; 95% CI: 1.3–3.1; *p* = 0.001), age >55 years (HR: 7.4; 95% CI: 4.5–12.1; *p* < 0.001), and RAI therapy (HR: 0.6; 95% CI: 0.4–1.0, *p* = 0.031) were independently associated with death from any cause. In the non‐low‐risk group, male sex was not independently associated with death from any cause. However, age >55 years (HR: 11.8; 95% CI: 7.4–18.9; *p* < 0.001), total thyroidectomy (HR: 1.8; 95% CI: 1.1–3.0; *p* = 0.005), RAI therapy (HR: 0.5; 95% CI: 0.3–0.7; *p* < 0.001), lateral lymph node metastasis (HR: 1.8; 95% CI: 1.2–2.8; *p* = 0.006), and distant metastasis at diagnosis (HR: 4.6; 95% CI: 2.3–9.3; *p* < 0.001) were independently associated with all‐cause mortality (Table 3).

**TABLE 3:** Univariate and multivariate Cox regression analysis for death from any cause.

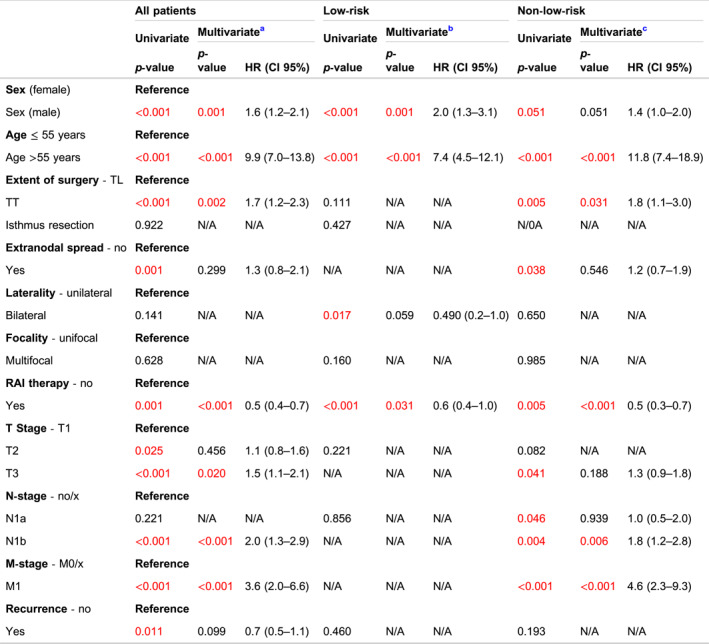

Abbreviation: HR, hazard ratio; TL, thyroid lobectomy, TT, total thyroidectomy.

^a^ Corrected for male sex, age >55 years, TT, present extranodal spread, RAI therapy, T2‐stage, T3‐stage, N1b‐stage, M1‐stage and recurrence.

^b^ Corrected for male sex, age >55 years, bilaterality and RAI therapy.

^c^ Corrected for male sex, age >55 years, TT, present extranodal spread, RAI therapy, T3‐stage, N1a‐stage, N1‐stage, and M1‐stage.

### 3.6 Risk of recurrence

**FIGURE 3:**
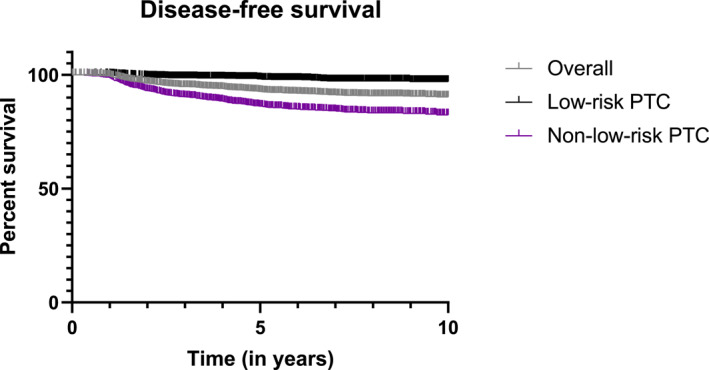
The 10‐year disease‐free survival in the low‐risk and non‐low‐risk Dutch papillary thyroid cancer patients.

During the follow‐up period, recurrence was observed in 257 patients (7.6%). Locoregional recurrence occurred in 239 (7.1%) patients, while distant recurrence occurred in 16 patients (0.5%). Only 6 patients (0.2%) progressed and developed distant metastases after locoregional recurrence. Recurrence rates were significantly higher in the non‐low‐risk group compared to the low‐risk group (14.0% vs. 2.2%, *p* < 0.001). Following primary surgery, 69 patients (2.0%) underwent additional unilateral neck dissection, and 12 patients underwent bilateral neck dissection for the recurrent disease. The median time until recurrence was 5.4 years (IQR: 3.4–8.4), with a longer duration observed in the low‐risk group compared to the non‐low‐risk group (5.7 vs. 5.1 years, *p* < 0.001). The 10‐year DFI for the overall group (excluding patients with distant metastasis at diagnosis) was 90.3%. Specifically, the 10‐year DFI was 97.0% in the low‐risk group and 82.4% in the non‐low‐risk group (*p* < 0.001) (Figure 3). multivariable Cox regression analysis revealed that age >55 years (HR: 2.1; 95% CI: 1.6–2.7; *p* < 0.001), RAI therapy (HR: 1.9; 95% CI: 1.2–3.1; *p* = 0.011), pT3 stage (HR: 2.0; 95% CI: 1.6–2.6; *p* < 0.001), central (HR: 2.5; 95% CI: 1.7–3.7; *p* < 0.001), and lateral lymph node metastasis (HR: 4.8; 95% CI: 3.5–6.6; *p* < 0.001) remained independent factors that associated with recurrence (Table 4). In the low‐risk group, only central lymph node metastasis (HR: 2.9; 95% CI: 1.3–6.7; *p* = 0.010) was independently associated with recurrence. In the non‐low‐risk group, age >55 years (HR: 2.1; 95% CI: 1.6–2.8; *p* < 0.001) and lateral lymph node metastasis (HR: 2.3; 95% CI: 1.7–3.1; *p* < 0.001) were independently associated with recurrence (Table 4).

**TABLE 4:** Univariate and multivariate Cox regression analysis for recurrence.

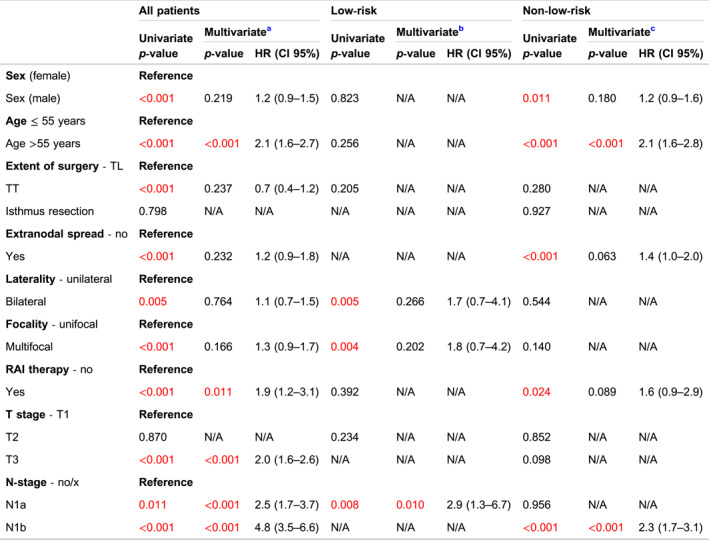

Abbreviation: HR, hazard ratio; TL, thyroid lobectomy, TT, total thyroidectomy.

^a^ Corrected for male sex, age >55 years, TT, present extranodal spread, bilaterality, multifocality, RAI therapy, T3‐stage, N1a‐stage and N1b‐stage.

^b^ Corrected for bilaterality, multifocality and N1a‐stage.

^c^ Corrected for male sex, age >55 years, present extranodal spread, RAI therapy and N1b‐stage.

### 3.7 Comparison of two national registries: Dutch PTC patients versus the U.S. patients

We have opted to compare the TNM stage between the Dutch and U.S. PTC population. Comparisons between the Dutch PTC population and published SEER data from U.S. cohorts are presented in Table 5. Compared to data from the SEER database and the National Cancer Database (NCDB), the Dutch PTC population had a lower frequency of pT1 tumors (45.8% vs. 58.2%–66.6%) and more pT2 (34.9% vs. 14.3%–22.0%) and the results concerning pT3 tumors were comparable (19.3% vs. 13.3%–27.5%).^11^, ^12^, ^13^, ^14^, ^15^, ^16^, ^17^, ^18^, ^19^ Also, the lateral lymph node metastases were more prevalent in the Dutch PTC population (16.6% vs. 7.4%–12.2%).^11^, ^13^ Distant metastases were comparable among both countries (0.5% vs. 0.8%–1.5%).^11^, ^12^, ^13^, ^14^, ^15^, ^16^, ^17^, ^18^, ^19^

**TABLE 5:** Comparisons between the DTC/papillary thyroid cancer populations.

	NCDB^11^	SEER^11^	SEER^12^	SEER^13^	SEER^14^	SEER^15^	SEER^16^	SEER^17^	SEER^18^	SEER^19^	Dutch population *n* = 3368
**Authors**	Orosco et al.	Orosco et al.	Chaves et al.	Liu et al.	Guo et al.	Liu et al.	Genpeng et al.	Zhang et al.	Oyer et al.	Barney et al.	Ten Hoor et al.
**Study population**	‐ DTC ‐ 2004–2012	‐ DTC ‐ 1992–2009	‐ PTC ‐ 1999–2008	‐ PTC ‐ 2006–2015	‐ PTC ‐ 2004–2017	‐ PTC ‐ 2004–2013	‐ PTC ‐ 2004–2015	‐ PTC ‐ 2004–2014	‐ PTC ‐ 1988–2003	‐ DTC ‐ 1983–2002	‐ PTC ‐ 2000–2015
**T‐stage ‐ no. (%)**	**n = 179,922**	**n = 65,797**	**n = 6982**	**n = 86,538**	**n = 94,979**	**n = 61,123**	**n = 31,495**	**n = 64,855**	**n = 31,646**	**n = 15,222**	**n = 3368**
T1	115,678 (64.3)	41,686 (63.4)	4063 (58.2)	57,600 (66.6)	58,333 (61.4)	39,520 (64.7)	20,419 (64.8)	42,231 (65.1)	19,259 (60.9)	8999 (59.1)	1541 (45.8)
T2	32,567 (18.1)	14,506 (22.0)	998 (14.3)	17,422 (20.1)	15,796 (16.6)	10,257 (16.8)	5723 (18.2)	10,661 (16.4)	6783 (21.4)	3193 (21.0)	1176 (34.9)
T3	31,677 (17.6)	9605 (14.6)	1921 (27.5)	11,516 (13.3)	20,850 (22.0)	11,346 (18.6)	5353 (17.0)	11,963 (18.4)	5604 (17.7)	3030 (19.9)	651 (19.3)
**N‐stage ‐ no. (%)**	**n = 174,509**	**n = 64,296**	**n = 8170**	**n = 89,204**	**n = 98,288**	**n = 63,219**	**n = 32,265**	**n = 69,034**	**n = 33,534**	**n = 18,415**	**n = 3368**
N1a	17,118 (9.8)	4286 (6.7)	‐	13,197 (14.8)	–	–	–	–	–	–	342 (10.2)
N1b	15,940 (9.1)	8029 (12.5)	‐	6624 (7.4)	–	–	–	–	–	–	560 (16.6)
N1 (location unknown)	–	–	1489 (18.2)	1660 (1.9)	26,471 (26.9)	14,292 (22.6)	4477 (13.9)	15,427 (22.3)	7447 (19.5)	3905 (21.2)	–
N0/unknown	141,451 (81.1)	51,981 (80.8)	6681 (81.8)	67,723 (75.9)	71,817 (73.1)	48,927 (77.4)	27,788 (86.1)	53,607 (77.6)	26,087 (98.2)	14,510 (78.8)	2466 (73.2)
**M‐stage ‐ no. (%)**	**n = 199,371**	**n = 77,187**	**n = 8170**	‐	**n = 98,288**	**n = 63,219**	**n = 32,265**	**n = 69,034**	**n = 33,534**	**n = 18,415**	**n = 3368**
M1	2139 (1.1)	992 (1.3)	198 (2.4)	‐	821 (0.8)	492 (0.8)	270 (0.8)	599 (0.9)	615 (1.8)	280 (1.5)	18 (0.5)
M0/unknown	197,232 (98.9)	76,195 (98.7)	7972 (97.6)	‐	97,467 (99.2)	62,727 (99.2)	31,995 (99.2)	68,435 (99.1)	32,919 (98.2)	18,135 (98.5)	3350 (99.5)

Abbreviation: DTC, differentiated thyroid cancer; NCDB, National Cancer Database, SEER, Surveillance Epidemiology and End Results.

## 4 DISCUSSION

This is the first study to classify the Dutch PTC population in low‐ and non‐low‐risk PTC patients based on the ATA risk stratification and to perform a literature review to compare the TNM stage of Dutch and U.S. PTC patients. These cohorts were compared because population‐based recommendations (such as the 2015 ATA de‐escalation recommendation to perform a hemithyroidectomy instead of a total thyroidectomy) from a particular country are often extrapolated to other countries, while the composition of the patient populations may not be directly comparable due to different diagnostic work‐ups. In the Dutch pT1‐T3 PTC patients diagnosed between 2005 and 2015, we found that pT1 tumors were most prevalent (45.8%), followed by pT2 (34.9%) and pT3 tumors (19.3%). Compared to the U.S. population (58.2%–66.6%), the proportion of pT1 tumors was lower.^11^, ^12^, ^13^, ^14^, ^15^, ^16^, ^17^, ^18^, ^19^ However, the proportion of pT2 tumors (34.9% vs. 14.3%–22.0%) was higher in the Dutch population. The relative incidence of pT3 tumors (19.3% vs. 13.3%–27.5%) was comparable (Table 5).^11^, ^12^, ^13^, ^14^, ^15^, ^16^, ^17^, ^18^, ^19^ Notably, lateral lymph node metastases (16.6% vs. 7.4%–12.2%) were found more frequently in Dutch patients compared to U.S. patients (Table 5).^11^, ^13^ Distant metastases at diagnosis were comparable in the Dutch PTC population (0.5%) compared to the U.S. population (0.5% vs. 0.8%–1.5%).^11^, ^12^, ^13^, ^14^, ^15^, ^16^, ^17^, ^18^, ^19^ We hypothesized that the abovementioned differences are probably attributable to a more restrictive diagnostic approach in the Netherlands.^20^

This hypothesis is supported by studies comparing PTC cohorts from neighboring countries and even within countries. A study by *Van Velsen et al.* comparing Dutch and German PTC patients found that lymph node metastases and distant metastases occurred more frequently in Dutch patients with a more restrictive diagnostic work‐up.^21^ Similarly, *Decallonne et al.* demonstrated that interregional differences in diagnostic practices influence the patient population, with the region employing higher rates of imaging modalities and surgical interventions, and less use of presurgical FNA (suggestive of a lower threshold for surgery) showing higher incidences of low‐risk pT1 thyroid tumors.^9^

A few limitations must be considered. Firstly, the recurrence rates of this study might be underestimated because only histologically or cytologically proven recurrences were registered, and biochemical recurrences are not registered in our national cancer registry. Recurrence could have been defined as locoregional recurrence or distant metastasis after 12 disease‐free months after RAI therapy, but unfortunately we had no dates of administration. Data on the cause of death was lacking, and therefore, all‐cause mortality was used which may render the results regarding the predictors of mortality less informative. However, since the disease recurrence rate is low and relapses can occur after many decades, the recurrence rate was regarded more clinically relevant. Furthermore, there was no data on the size of the lymph node metastases, which is needed to further nuance the ATA risk stratification. Additionally, limited data on tumor biology of the PTCs was available.^22^, ^23^ Lastly, this study is limited by its retrospective nature and the fact that the data is from 2005 to 2015. However, the content of the Dutch guidelines during these years and the subsequent years has been the same regarding the diagnostic work‐up.^6^

The 2015 ATA guidelines propose to de‐escalate surgical treatment for low‐risk PTC, advocating for a hemithyroidectomy rather than a total thyroidectomy for patients with 1–4 cm PTCs.^5^ However, after careful examination of the Dutch PTC population in our study, the suggestion arises that the applicability of this recommendation to the Dutch population may be more nuanced. It is feasible to perform a hemithyroidectomy in carefully selected low‐risk PTC patients, but the a priori chance of encountering these patients is low in the Dutch PTC population.^24^ Furthermore, it seems that because more incidentalomas (probably being low‐risk T1 tumors) are diagnosed in the U.S., diluting the patients outcomes for survival and recurrence, the counseling in the Netherlands for patients with T1 tumors should be based on the outcomes of our own national data. It also highlights that the diagnostic practices specific to a particular country or region should be taken into account when considering adopting a foreign recommendation and recurrence outcomes should be monitored.^5^, ^7^

The most optimal treatment for PTC is still a matter of international debate; however, national differences in diagnostic strategies PTC lead to different PTC patient populations that necessitate approaches being adapted to the national practices, advocating a data‐driven de‐escalation on a national basis.

## AUTHOR CONTRIBUTIONS

**Maaike B. C. Hoor**: Conceptualization; data curation; formal analysis; investigation; methodology; project administration; visualization; writing—original draft; writing—review & editing. **Jia F. Lin**: Conceptualization; data curation; formal analysis; methodology; supervision; writing—review & editing. **Madelon J. H. Metman**: Conceptualization; data curation; writing—review & editing. **Pedro M. Rodriguez Schaap**: Data curation; project administration; writing—review & editing. **Thera P. Links**: Conceptualization; methodology; writing—review & editing. **Renske Altena**: Supervision; writing—review & editing. **Tessa M. van Ginhoven**: Data curation; writing—review & editing. **Wouter T. Zandee**: Methodology; supervision; writing—review & editing. **Anton F. Engelsman**: Conceptualization; data curation; writing—review & editing. **Schelto Kruijff**: Conceptualization; investigation; methodology; project administration; supervision; writing—review & editing.

## CONFLICT OF INTEREST STATEMENT

The authors declare no conflicts of interest.

## FUNDING INFORMATION

None.

## ETHICS STATEMENT

This study was approved by the Amsterdam UMC medical ethical committee.

## Supporting information

Table S1

Table S2
