# Supplementary material for: Does a Restrictive Diagnostic Work‐up for Thyroid Nodules Lead to a Different Papillary Thyroid Cancer Patient Population? A Comparison Between Dutch and U.S. T1‐T3 Patient Population
Source: World J Surg. 2024 Dec 25;49(4):985–96. doi: 10.1002/wjs.12457 (PMC11994149; doi:10.1002/wjs.12457)
Supplement: Supplementary file 1 — Table S1 [file WJS-49-985-s002.docx]

**Supplementary table 1.** Systematic review of studies on the ratio between low-risk and non-low-risk PTC.

| **Authors** | **Inclusion period** | **Country** | **Patient population** | **Definition of low-risk** | **No. of patients** | **Low-risk**  **no. (%)** | **Non-low-risk**  **no. (%)** |
| --- | --- | --- | --- | --- | --- | --- | --- |
| Ten Hoor et al.  (this study) | 2005-2015 | The Netherlands | Inclusion: - PTC | In line with the risk-stratification of the 2015 ATA guidelines. | 3368 | 1808 (53.7) | 1560 (43.3) |
| Haigh et al.(9) | 1988-1995 | U.S. (SEER) | Inclusion: - PTC   Exclusion:  - ≤19 years  - missing data | - ≤40 years for men and ≤50 years for women, with intrathyroidal PTC - All older patients with intrathyroidal PTC <5cm without distant metastasis | 5432 | 4402 (81.0) | 1030 (19.0) |
| Yang et al.(10) | 2004-2014 | U.S. (SEER) | Inclusion : - mPTC | In line with the risk-stratification of the 2015 ATA guidelines. | 39 032 | 33,428 (85.6) | 5604 (14.4) |
| Zhao and Gong (11) | 2000-2018 | U.S. (SEER) | Inclusion: - PTC  Exclusion: - ≥2 tumours - <18 years - non-positive histology - chemotherapy - distant metastasis  - pT4 | In line with the risk-stratification of the 2015 ATA guidelines. | 6770 | 3779 (55.8) | 2991 (44.2) *(only intermediate)* |
| Song et al.(14) | 1996-2009 | South-Korea | Inclusion: - 1-4cm PTC - underwent lobectomy  Exclusion: - N1b-stage - distant metastasis - completion thyroidectomy after initial lobectomy | In line with the risk-stratification of the 2015 ATA guidelines. | 571 | 222 (38.9) | 349 (61.1) |
| Tuttle et al.(16) | 1994-2004 | U.S. | Inclusion: - DTC - total thyroidectomy and RAI therapy  Exclusion - inadequate data (FU, staging)  - Interfering anti-Tg antibodies - anaplastic thyroid cancer - <18 years at diagnosis | All are present: - No local or distant metastasis - Free surgical margins - No aggressive histology - No vascular invasion - No iodine uptake outside the thyroid bed on post-treatment scan | 588 | 135 (23.0) | 453 (77.0) |
| Vaisman et al.(15) | 1986-2009 | Brasil | Inclusion - DTC - total thyroidectomy and RAI therapy - ≥3 years of FU | In line with the risk-stratification of the 2015 ATA guidelines. | 506 | 168 (33.2) | 338 (66.8) |
| Castagna et al.(17) | Unknown | Italy | Inclusion: - DTC - Single-center | In line with the risk-stratification of the 2009 ATA guidelines. | 512 | 244 (47.6) | 268 (52.4) |
| Pitoia et al.(18) | 2001-2011 | Argentina | Inclusion: - DTC - total thyroidectomy and RAI therapy - Single-center | In line with the risk-stratification of the 2009 ATA guidelines. | 171 | 60 (35.1) | 111 (64.9) |
