# Supplementary material for: Does a Restrictive Diagnostic Work‐up for Thyroid Nodules Lead to a Different Papillary Thyroid Cancer Patient Population? A Comparison Between Dutch and U.S. T1‐T3 Patient Population
Source: World J Surg. 2024 Dec 25;49(4):985–96. doi: 10.1002/wjs.12457 (PMC11994149; doi:10.1002/wjs.12457)
Supplement: Supplementary file 2 — Table S2 [file WJS-49-985-s001.docx]

**Supplementary table 2**. Systematic review of the U.S. SEER data on DTC and PTC populations.

| **1. After analyzing the title (n=237)** |  |
| --- | --- |
| **Number of excluded manuscripts** | **Reason** |
| 3 | Other type of study (survey, comment) |
| 7 | Not in the U.S. (South-Korea, Hawaii, California and Saudi Arabia) |
| 21 | Different type of thyroid cancer than PTC or DTC (PTC and FTC) (pediatric, familial, spindle cell, only FTC, medullary) |
| 24 | Only within one sub-group of the population (women, age-groups, military personnel, untreated/refused treatment) |
| 28 | Only within one sub-group of PTC patients (based on TNM-stage, grade, risk-stratification, stage, who underwent different types of neck dissections) |
| 42 | Focused on specific subtypes of PTC (insular, tall cell, columnar cell, microcarcinoma, follicular variant, mixed subtype, aggressive variants, Hürthle cell, diffuse sclerosing) |
| 13 | About different cancers or diseases than PTC/DTC (breast, endocrine, merkel cell, intraductal papillary breast cancer, kidney and renal, DICER 1 syndrome) |
| 12 | About different subjects, no mentioning of TNM-stage (procedure guideline, fine needle aspiration, secondary malignancies, coeliac disease, social vulnerability, skeletal complications) |
| 1 | Duplicate |
| Total | 151 |
| **2. After reading the abstract** |  |
| 46 | No complete TNM-stage mention |
| 4 | No SEER data |
| 4 | All thyroid cancers, not concentrated on PTC or DTC |
| 13 | Only thyroid cancer in general |
| 1 | Endocrine cancers in general |
| Total | 68 |
| **3. After reading the manuscript in full** |  |
| 6 | No complete T-stage and N-stage |
| 1 | No concentration on PTC or DTC |
| 1 | T3 and T4 only together analyzed |
| 1 | Only patients with distant metastasis on presentation |
| Total | 9 |
| **Remaining manuscripts** | **9** |
